# Supplementary material for: Establishment of an in vivo analytical method for detecting total anti-UFH activity and pharmacokinetic study in PS and R15 in rats
Source: PLoS One. 2025 Oct 7;20(10):e0333619. doi: 10.1371/journal.pone.0333619 (PMC12503259; doi:10.1371/journal.pone.0333619)
Supplement: S1 File — S1 Table. Standard curve of PS in blank plasma. S2 Table. Standard curve of R15 in blank plasma. S3 Table. The stability of PS plasma sample placed in room temperature (25°C) for 30 min (n = 6). S4 Table. The stability of PS plasma sample freeze-thaw three cycles in −20°C (n = 6). S5 Table. The stability of stock solution of PS for 1 week (n = 6). S6 Table. The stability of R15 plasma sample placed in room temperature (25°C) for 30 min (n = 6). S7 Table. The stability of R15 plasma sample freeze-thaw three cycles in −20°C (n = 6). S8 Table. The stability of stock solution of R15 for 1 week (n = 6). S9 Table. Dilution effects of varying concentrations of plasma samples of PS diluted 2-fold, 5-fold, 10-fold, 20-fold (n = 5). S10 Table. Dilution effects of varying concentrations of plasma samples of R15 diluted 2-fold or 100-fold (n = 5). S11 Table. Pharmacokinetic parameters of intravenous infusion administration with PS (300 U/kg) to individual Wistar rats (n = 6). S11 Table. Pharmacokinetic parameters of intravenous infusion administration with PS (300 U/kg) to individual Wistar rats (n = 6). S12 Table. The plasma concentration of PS after intravenous infusion administration with PS (300 U/kg) to individual Wistar rats. ND: Not determined. S13 Table. Pharmacokinetic parameters of intravenous infusion administration with R15 (2700 U/kg) to individual Wistar rats (n = 8). S14 Table. Pharmacokinetic parameters of intravenous infusion administration with R15 (900 U/kg) to individual Wistar rats (n = 8). S15 Table. Pharmacokinetic parameters of intravenous infusion administration with R15 (300 U/kg) to individual Wistar rats (n = 8). S16 Table. The plasma concentration of R15 after intravenous infusion administration with R15 (300 U/kg) to individual Wistar rats. ND: Not determined. S17 Table. The plasma concentration of R15 after intravenous infusion administration with R15 (900 U/kg) to individual Wistar rats. ND: Not determined. S18 Table. The plasma concentration of [file pone.0333619.s001.zip › S File/S11_File.docx]

**S11 Table. Pharmacokinetic parameters of intravenous infusion administration with PS（300 U/kg）to individual Wistar rats (n=6)**

| **Parameter (Units)** | **PS（300 U/kg）** | | | | | | | | **Mean±SD** |
| --- | --- | --- | --- | --- | --- | --- | --- | --- | --- |
|  | **1#** | **2#** | **3#** | **6#** | **11#** | **12#** | **13#** | **14#** |  |
| T_1/2_ (min) | 1.72 | 7.06 | 0.00 | 8.65 | 1.64 | 2.18 | 2.41 | 1.20 | 3.94±3.08 |
| C_max_ (µg･mL^-1^) | 12.10 | 15.14 | 11.17 | 14.12 | 12.55 | 12.70 | 9.40 | 10.34 | 12.67±1.96 |
| AUC (min･µg･mL^-1^) | 35.86 | 42.97 | 17.83 | 45.62 | 29.16 | 33.06 | 28.68 | 18.80 | 35.89±7.07 |
| V_d_ (mL･kg^-1^) | 134 | 425 | 0 | 489 | 156 | 173 | 229 | 168 | 268±151 |
| CL (mL･min^-1^･kg^-1^) | 53.74 | 41.70 | 0.00 | 39.21 | 66.07 | 55.19 | 66.01 | 97.21 | 53.65±11.49 |
| MRT (min) | 2.51 | 3.20 | 1.12 | 4.40 | 2.12 | 2.49 | 2.97 | 1.46 | 2.95±0.81 |
