# Supplementary material for: Establishment of an in vivo analytical method for detecting total anti-UFH activity and pharmacokinetic study in PS and R15 in rats
Source: PLoS One. 2025 Oct 7;20(10):e0333619. doi: 10.1371/journal.pone.0333619 (PMC12503259; doi:10.1371/journal.pone.0333619)
Supplement: S1 File — S1 Table. Standard curve of PS in blank plasma. S2 Table. Standard curve of R15 in blank plasma. S3 Table. The stability of PS plasma sample placed in room temperature (25°C) for 30 min (n = 6). S4 Table. The stability of PS plasma sample freeze-thaw three cycles in −20°C (n = 6). S5 Table. The stability of stock solution of PS for 1 week (n = 6). S6 Table. The stability of R15 plasma sample placed in room temperature (25°C) for 30 min (n = 6). S7 Table. The stability of R15 plasma sample freeze-thaw three cycles in −20°C (n = 6). S8 Table. The stability of stock solution of R15 for 1 week (n = 6). S9 Table. Dilution effects of varying concentrations of plasma samples of PS diluted 2-fold, 5-fold, 10-fold, 20-fold (n = 5). S10 Table. Dilution effects of varying concentrations of plasma samples of R15 diluted 2-fold or 100-fold (n = 5). S11 Table. Pharmacokinetic parameters of intravenous infusion administration with PS (300 U/kg) to individual Wistar rats (n = 6). S11 Table. Pharmacokinetic parameters of intravenous infusion administration with PS (300 U/kg) to individual Wistar rats (n = 6). S12 Table. The plasma concentration of PS after intravenous infusion administration with PS (300 U/kg) to individual Wistar rats. ND: Not determined. S13 Table. Pharmacokinetic parameters of intravenous infusion administration with R15 (2700 U/kg) to individual Wistar rats (n = 8). S14 Table. Pharmacokinetic parameters of intravenous infusion administration with R15 (900 U/kg) to individual Wistar rats (n = 8). S15 Table. Pharmacokinetic parameters of intravenous infusion administration with R15 (300 U/kg) to individual Wistar rats (n = 8). S16 Table. The plasma concentration of R15 after intravenous infusion administration with R15 (300 U/kg) to individual Wistar rats. ND: Not determined. S17 Table. The plasma concentration of R15 after intravenous infusion administration with R15 (900 U/kg) to individual Wistar rats. ND: Not determined. S18 Table. The plasma concentration of [file pone.0333619.s001.zip › S File/S12_File.docx]

**S12 Table. The plasma concentration of PS after intravenous infusion administration with PS（300 U/kg）to individual Wistar rats**

| **Time (min)** | **Concentration (μg/mL)** | | | | | | | | Mean±SD |
| --- | --- | --- | --- | --- | --- | --- | --- | --- | --- |
|  | **1#** | **2#** | **3#** | **6#** | **11#** | **12#** | **13#** | **14#** |  |
| 0 | 0.00 | 0.00 | 0.00 | 0.00 | 0.00 | 0.00 | 0.00 | 0.00 | 0.00 |
| 1 | 12.10 | 15.14 | 11.17 | 14.12 | 12.55 | 12.70 | 9.40 | 10.34 | 12.19±1.89 |
| 3 | 5.66 | 4.67 | 1.07 | 4.32 | 3.14 | 3.65 | 3.65 | 1.13 | 3.41±1.62 |
| 5 | 2.03 | 2.20 | ND | 2.12 | 1.32 | 1.87 | 1.73 | 1.03 | 1.76±0.44 |
| 7 | 0.90 | 0.87 | ND | 1.37 | 0.48 | 0.98 | 0.83 | ND | 0.91±0.29 |
| 9 | 0.54 | 0.90 | ND | 1.13 | 0.47 | 1.01 | 0.84 | ND | 0.82±0.26 |
| 11 | ND | 0.66 | ND | 0.89 | ND | ND | 0.47 | ND | 0.67±0.21 |
| 15 | ND | 0.49 | ND | 0.62 | ND | ND | ND | ND | 0.55±0.09 |
| 20 | ND | ND | ND | 0.43 | ND | ND | ND | ND | 0.43 |
| 30 | ND | ND | ND | ND | ND | ND | ND | ND | / |
| 60 | ND | ND | ND | ND | ND | ND | ND | ND | / |
| 120 | ND | ND | ND | ND | ND | ND | ND | ND | / |

ND：Not determined
