# Supplementary material for: Establishment of an in vivo analytical method for detecting total anti-UFH activity and pharmacokinetic study in PS and R15 in rats
Source: PLoS One. 2025 Oct 7;20(10):e0333619. doi: 10.1371/journal.pone.0333619 (PMC12503259; doi:10.1371/journal.pone.0333619)
Supplement: S1 File — S1 Table. Standard curve of PS in blank plasma. S2 Table. Standard curve of R15 in blank plasma. S3 Table. The stability of PS plasma sample placed in room temperature (25°C) for 30 min (n = 6). S4 Table. The stability of PS plasma sample freeze-thaw three cycles in −20°C (n = 6). S5 Table. The stability of stock solution of PS for 1 week (n = 6). S6 Table. The stability of R15 plasma sample placed in room temperature (25°C) for 30 min (n = 6). S7 Table. The stability of R15 plasma sample freeze-thaw three cycles in −20°C (n = 6). S8 Table. The stability of stock solution of R15 for 1 week (n = 6). S9 Table. Dilution effects of varying concentrations of plasma samples of PS diluted 2-fold, 5-fold, 10-fold, 20-fold (n = 5). S10 Table. Dilution effects of varying concentrations of plasma samples of R15 diluted 2-fold or 100-fold (n = 5). S11 Table. Pharmacokinetic parameters of intravenous infusion administration with PS (300 U/kg) to individual Wistar rats (n = 6). S11 Table. Pharmacokinetic parameters of intravenous infusion administration with PS (300 U/kg) to individual Wistar rats (n = 6). S12 Table. The plasma concentration of PS after intravenous infusion administration with PS (300 U/kg) to individual Wistar rats. ND: Not determined. S13 Table. Pharmacokinetic parameters of intravenous infusion administration with R15 (2700 U/kg) to individual Wistar rats (n = 8). S14 Table. Pharmacokinetic parameters of intravenous infusion administration with R15 (900 U/kg) to individual Wistar rats (n = 8). S15 Table. Pharmacokinetic parameters of intravenous infusion administration with R15 (300 U/kg) to individual Wistar rats (n = 8). S16 Table. The plasma concentration of R15 after intravenous infusion administration with R15 (300 U/kg) to individual Wistar rats. ND: Not determined. S17 Table. The plasma concentration of R15 after intravenous infusion administration with R15 (900 U/kg) to individual Wistar rats. ND: Not determined. S18 Table. The plasma concentration of [file pone.0333619.s001.zip › S File/S13_File.docx]

**S13 Table. Pharmacokinetic parameters of intravenous infusion administration with R15（2700 U/kg）to individual Wistar rats (n=8)**

| **Parameter (Units)** | **R15（2700 U/kg）** | | | | | | | | **Mean±SD** |
| --- | --- | --- | --- | --- | --- | --- | --- | --- | --- |
|  | **1#** | **5#** | **8#** | **13#** | **21#** | **22#** | **27#** | **28#** |  |
| T_1/2_ (min) | 116.81 | 148.68 | 97.21 | 244.86 | 127.87 | 192.92 | 143.57 | 233.01 | 163.12±54.48 |
| C_max_ (µg･mL^-1^) | 14.61 | 10.94 | 12.70 | 13.51 | 15.63 | 8.27 | 7.46 | 10.11 | 11.65±2.95 |
| AUC (min･µg･mL^-1^) | 1918 | 654 | 1251 | 1141 | 1674 | 1224 | 1156 | 1543 | 1320±387 |
| V_d_ (mL･kg^-1^) | 1341 | 3477 | 1684 | 4129 | 1667 | 3091 | 2543 | 3015 | 2618±987 |
| CL (mL･min^-1^･kg^-1^) | 7.96 | 16.21 | 12.01 | 11.69 | 9.03 | 11.11 | 12.28 | 8.97 | 11.16±2.60 |
| MRT (min) | 239.44 | 96.70 | 172.95 | 218.22 | 235.05 | 223.18 | 144.44 | 220.97 | 193.87±51.11 |
