# Supplementary material for: Establishment of an in vivo analytical method for detecting total anti-UFH activity and pharmacokinetic study in PS and R15 in rats
Source: PLoS One. 2025 Oct 7;20(10):e0333619. doi: 10.1371/journal.pone.0333619 (PMC12503259; doi:10.1371/journal.pone.0333619)
Supplement: S1 File — S1 Table. Standard curve of PS in blank plasma. S2 Table. Standard curve of R15 in blank plasma. S3 Table. The stability of PS plasma sample placed in room temperature (25°C) for 30 min (n = 6). S4 Table. The stability of PS plasma sample freeze-thaw three cycles in −20°C (n = 6). S5 Table. The stability of stock solution of PS for 1 week (n = 6). S6 Table. The stability of R15 plasma sample placed in room temperature (25°C) for 30 min (n = 6). S7 Table. The stability of R15 plasma sample freeze-thaw three cycles in −20°C (n = 6). S8 Table. The stability of stock solution of R15 for 1 week (n = 6). S9 Table. Dilution effects of varying concentrations of plasma samples of PS diluted 2-fold, 5-fold, 10-fold, 20-fold (n = 5). S10 Table. Dilution effects of varying concentrations of plasma samples of R15 diluted 2-fold or 100-fold (n = 5). S11 Table. Pharmacokinetic parameters of intravenous infusion administration with PS (300 U/kg) to individual Wistar rats (n = 6). S11 Table. Pharmacokinetic parameters of intravenous infusion administration with PS (300 U/kg) to individual Wistar rats (n = 6). S12 Table. The plasma concentration of PS after intravenous infusion administration with PS (300 U/kg) to individual Wistar rats. ND: Not determined. S13 Table. Pharmacokinetic parameters of intravenous infusion administration with R15 (2700 U/kg) to individual Wistar rats (n = 8). S14 Table. Pharmacokinetic parameters of intravenous infusion administration with R15 (900 U/kg) to individual Wistar rats (n = 8). S15 Table. Pharmacokinetic parameters of intravenous infusion administration with R15 (300 U/kg) to individual Wistar rats (n = 8). S16 Table. The plasma concentration of R15 after intravenous infusion administration with R15 (300 U/kg) to individual Wistar rats. ND: Not determined. S17 Table. The plasma concentration of R15 after intravenous infusion administration with R15 (900 U/kg) to individual Wistar rats. ND: Not determined. S18 Table. The plasma concentration of [file pone.0333619.s001.zip › S File/S14_File.docx]

**S14 Table. Pharmacokinetic parameters of intravenous infusion administration with R15（900 U/kg）to individual Wistar rats (n=8)**

| **Parameter (Units)** | **R15（900 U/kg）** | | | | | | | | **Mean±SD** |
| --- | --- | --- | --- | --- | --- | --- | --- | --- | --- |
|  | **2#** | **3#** | **4#** | **12#** | **16#** | **23#** | **24#** | **26#** |  |
| T_1/2_ (min) | 91.11 | 118.30 | 75.46 | 71.74 | 100.73 | 95.83 | 76.10 | 99.79 | 91.13±15.92 |
| C_max_ (µg･mL^-1^) | 10.95 | 10.91 | 10.42 | 13.54 | 11.60 | 10.72 | 13.04 | 11.10 | 11.54±1.14 |
| AUC (min･µg･mL^-1^) | 1062 | 1265 | 805 | 862 | 1036 | 1297 | 906 | 1011 | 1030±178 |
| V_d_ (mL･kg^-1^) | 563 | 675 | 652 | 574 | 613 | 518 | 581 | 685 | 608±59 |
| CL (mL･min^-1^･kg^-1^) | 4.28 | 3.96 | 5.99 | 5.55 | 4.22 | 3.74 | 5.29 | 4.76 | 4.72±0.81 |
| MRT (min) | 82.99 | 136.48 | 77.98 | 78.56 | 83.90 | 113.45 | 77.69 | 104.14 | 94.40±21.61 |
