# Supplementary material for: Establishment of an in vivo analytical method for detecting total anti-UFH activity and pharmacokinetic study in PS and R15 in rats
Source: PLoS One. 2025 Oct 7;20(10):e0333619. doi: 10.1371/journal.pone.0333619 (PMC12503259; doi:10.1371/journal.pone.0333619)
Supplement: S1 File — S1 Table. Standard curve of PS in blank plasma. S2 Table. Standard curve of R15 in blank plasma. S3 Table. The stability of PS plasma sample placed in room temperature (25°C) for 30 min (n = 6). S4 Table. The stability of PS plasma sample freeze-thaw three cycles in −20°C (n = 6). S5 Table. The stability of stock solution of PS for 1 week (n = 6). S6 Table. The stability of R15 plasma sample placed in room temperature (25°C) for 30 min (n = 6). S7 Table. The stability of R15 plasma sample freeze-thaw three cycles in −20°C (n = 6). S8 Table. The stability of stock solution of R15 for 1 week (n = 6). S9 Table. Dilution effects of varying concentrations of plasma samples of PS diluted 2-fold, 5-fold, 10-fold, 20-fold (n = 5). S10 Table. Dilution effects of varying concentrations of plasma samples of R15 diluted 2-fold or 100-fold (n = 5). S11 Table. Pharmacokinetic parameters of intravenous infusion administration with PS (300 U/kg) to individual Wistar rats (n = 6). S11 Table. Pharmacokinetic parameters of intravenous infusion administration with PS (300 U/kg) to individual Wistar rats (n = 6). S12 Table. The plasma concentration of PS after intravenous infusion administration with PS (300 U/kg) to individual Wistar rats. ND: Not determined. S13 Table. Pharmacokinetic parameters of intravenous infusion administration with R15 (2700 U/kg) to individual Wistar rats (n = 8). S14 Table. Pharmacokinetic parameters of intravenous infusion administration with R15 (900 U/kg) to individual Wistar rats (n = 8). S15 Table. Pharmacokinetic parameters of intravenous infusion administration with R15 (300 U/kg) to individual Wistar rats (n = 8). S16 Table. The plasma concentration of R15 after intravenous infusion administration with R15 (300 U/kg) to individual Wistar rats. ND: Not determined. S17 Table. The plasma concentration of R15 after intravenous infusion administration with R15 (900 U/kg) to individual Wistar rats. ND: Not determined. S18 Table. The plasma concentration of [file pone.0333619.s001.zip › S File/S15_File.docx]

**S15 Table. Pharmacokinetic parameters of intravenous infusion administration with R15 (300 U/kg) to individual Wistar rats (n=8)**

| **Parameter (Units)** | **R15（300 U/kg）** | | | | | | | | **Mean±SD** |
| --- | --- | --- | --- | --- | --- | --- | --- | --- | --- |
|  | **9#** | **11#** | **14#** | **15#** | **18#** | **19#** | **20#** | **30#** |  |
| T_1/2_ (min) | 63.73 | 46.57 | 32.56 | 37.28 | 63.84 | 60.35 | 61.73 | 49.35 | 51.93±12.38 |
| C_max_ (µg･mL^-1^) | 9.90 | 10.65 | 8.02 | 7.14 | 13.09 | 12.60 | 14.00 | 11.67 | 10.89±2.43 |
| AUC (min･µg･mL^-1^) | 662 | 427 | 423 | 452 | 753 | 757 | 920 | 665 | 632±182 |
| V_d_ (mL･kg^-1^) | 225 | 231 | 180 | 187 | 199 | 189 | 160 | 182 | 194±24 |
| CL (mL･min^-1^･kg^-1^) | 2.45 | 3.44 | 3.83 | 3.47 | 2.16 | 2.17 | 1.79 | 2.56 | 2.73±0.75 |
| MRT (min) | 65.78 | 41.02 | 39.64 | 40.49 | 64.13 | 62.54 | 67.99 | 57.96 | 54.95±12.40 |
