# Supplementary material for: Establishment of an in vivo analytical method for detecting total anti-UFH activity and pharmacokinetic study in PS and R15 in rats
Source: PLoS One. 2025 Oct 7;20(10):e0333619. doi: 10.1371/journal.pone.0333619 (PMC12503259; doi:10.1371/journal.pone.0333619)
Supplement: S1 File — S1 Table. Standard curve of PS in blank plasma. S2 Table. Standard curve of R15 in blank plasma. S3 Table. The stability of PS plasma sample placed in room temperature (25°C) for 30 min (n = 6). S4 Table. The stability of PS plasma sample freeze-thaw three cycles in −20°C (n = 6). S5 Table. The stability of stock solution of PS for 1 week (n = 6). S6 Table. The stability of R15 plasma sample placed in room temperature (25°C) for 30 min (n = 6). S7 Table. The stability of R15 plasma sample freeze-thaw three cycles in −20°C (n = 6). S8 Table. The stability of stock solution of R15 for 1 week (n = 6). S9 Table. Dilution effects of varying concentrations of plasma samples of PS diluted 2-fold, 5-fold, 10-fold, 20-fold (n = 5). S10 Table. Dilution effects of varying concentrations of plasma samples of R15 diluted 2-fold or 100-fold (n = 5). S11 Table. Pharmacokinetic parameters of intravenous infusion administration with PS (300 U/kg) to individual Wistar rats (n = 6). S11 Table. Pharmacokinetic parameters of intravenous infusion administration with PS (300 U/kg) to individual Wistar rats (n = 6). S12 Table. The plasma concentration of PS after intravenous infusion administration with PS (300 U/kg) to individual Wistar rats. ND: Not determined. S13 Table. Pharmacokinetic parameters of intravenous infusion administration with R15 (2700 U/kg) to individual Wistar rats (n = 8). S14 Table. Pharmacokinetic parameters of intravenous infusion administration with R15 (900 U/kg) to individual Wistar rats (n = 8). S15 Table. Pharmacokinetic parameters of intravenous infusion administration with R15 (300 U/kg) to individual Wistar rats (n = 8). S16 Table. The plasma concentration of R15 after intravenous infusion administration with R15 (300 U/kg) to individual Wistar rats. ND: Not determined. S17 Table. The plasma concentration of R15 after intravenous infusion administration with R15 (900 U/kg) to individual Wistar rats. ND: Not determined. S18 Table. The plasma concentration of [file pone.0333619.s001.zip › S File/S16_File.docx]

**S16 Table. The plasma concentration of R15 after intravenous infusion administration with R15 (300 U/kg) to individual Wistar rats**

| **Time (min)** | **Concentration (μg/mL)** | | | | | | | | **Mean±SD** |
| --- | --- | --- | --- | --- | --- | --- | --- | --- | --- |
|  | **9#** | **11#** | **14#** | **15#** | **18#** | **19#** | **20#** | **30#** |  |
| 0 | 0.00 | 0.00 | 0.00 | 0.00 | 0.00 | 0.00 | 0.00 | 0.00 | 0.00 |
| 1 | 9.90 | 10.65 | 8.02 | 7.14 | 13.09 | 12.60 | 14.00 | 11.67 | 10.89±2.43 |
| 5 | 7.50 | 5.50 | 4.84 | 6.81 | 7.78 | 8.79 | 7.80 | 6.89 | 6.99±1.29 |
| 15 | 7.19 | 6.01 | 6.23 | 6.00 | 8.06 | 7.83 | 9.23 | 8.14 | 7.34±1.18 |
| 30 | 5.91 | 4.92 | 5.19 | 5.39 | 6.03 | 6.34 | 7.22 | 6.04 | 5.88±0.73 |
| 60 | 3.72 | 3.06 | 3.36 | 3.53 | 5.28 | 5.28 | 5.90 | 4.38 | 4.31±1.06 |
| 120 | 1.74 | 1.27 | 0.80 | 1.04 | 1.65 | 1.64 | 2.70 | 1.46 | 1.54±0.57 |
| 240 | 0.63 | ND | ND | ND | 0.71 | 0.65 | 0.73 | 0.35 | 0.61±0.15 |
| 360 | ND | ND | ND | ND | ND | ND | ND | ND | / |
| 480 | ND | ND | ND | ND | ND | ND | ND | ND | / |

ND：Not determined
