# Supplementary material for: Establishment of an in vivo analytical method for detecting total anti-UFH activity and pharmacokinetic study in PS and R15 in rats
Source: PLoS One. 2025 Oct 7;20(10):e0333619. doi: 10.1371/journal.pone.0333619 (PMC12503259; doi:10.1371/journal.pone.0333619)
Supplement: S1 File — S1 Table. Standard curve of PS in blank plasma. S2 Table. Standard curve of R15 in blank plasma. S3 Table. The stability of PS plasma sample placed in room temperature (25°C) for 30 min (n = 6). S4 Table. The stability of PS plasma sample freeze-thaw three cycles in −20°C (n = 6). S5 Table. The stability of stock solution of PS for 1 week (n = 6). S6 Table. The stability of R15 plasma sample placed in room temperature (25°C) for 30 min (n = 6). S7 Table. The stability of R15 plasma sample freeze-thaw three cycles in −20°C (n = 6). S8 Table. The stability of stock solution of R15 for 1 week (n = 6). S9 Table. Dilution effects of varying concentrations of plasma samples of PS diluted 2-fold, 5-fold, 10-fold, 20-fold (n = 5). S10 Table. Dilution effects of varying concentrations of plasma samples of R15 diluted 2-fold or 100-fold (n = 5). S11 Table. Pharmacokinetic parameters of intravenous infusion administration with PS (300 U/kg) to individual Wistar rats (n = 6). S11 Table. Pharmacokinetic parameters of intravenous infusion administration with PS (300 U/kg) to individual Wistar rats (n = 6). S12 Table. The plasma concentration of PS after intravenous infusion administration with PS (300 U/kg) to individual Wistar rats. ND: Not determined. S13 Table. Pharmacokinetic parameters of intravenous infusion administration with R15 (2700 U/kg) to individual Wistar rats (n = 8). S14 Table. Pharmacokinetic parameters of intravenous infusion administration with R15 (900 U/kg) to individual Wistar rats (n = 8). S15 Table. Pharmacokinetic parameters of intravenous infusion administration with R15 (300 U/kg) to individual Wistar rats (n = 8). S16 Table. The plasma concentration of R15 after intravenous infusion administration with R15 (300 U/kg) to individual Wistar rats. ND: Not determined. S17 Table. The plasma concentration of R15 after intravenous infusion administration with R15 (900 U/kg) to individual Wistar rats. ND: Not determined. S18 Table. The plasma concentration of [file pone.0333619.s001.zip › S File/S17_File.docx]

**S17 Table. The plasma concentration of R15 after intravenous infusion administration with R15 (900 U/kg) to individual Wistar rats**

| **Time (min)** | **Concentration (μg/mL)** | | | | | | | | **Mean±SD** |
| --- | --- | --- | --- | --- | --- | --- | --- | --- | --- |
|  | **2#** | **3#** | **4#** | **12#** | **16#** | **23#** | **24#** | **26#** |  |
| 0 | 0.00 | 0.00 | 0.00 | 0.00 | 0.00 | 0.00 | 0.00 | 0.00 | 0.00 |
| 1 | 10.95 | 10.91 | 10.42 | 13.54 | 11.60 | 10.72 | 13.04 | 11.10 | 11.54±1.14 |
| 5 | 8.54 | 8.38 | 6.66 | 7.19 | 9.04 | 8.50 | 8.68 | 8.22 | 8.15±0.81 |
| 15 | 7.87 | 7.38 | 6.90 | 6.72 | 7.26 | 7.32 | 7.22 | 7.16 | 7.23±0.34 |
| 30 | 6.65 | 5.58 | 4.79 | 5.08 | 6.57 | 5.73 | 5.44 | 5.67 | 5.69±0.65 |
| 60 | 5.51 | 4.94 | 4.37 | 4.91 | 5.44 | 6.22 | 4.89 | 4.56 | 5.11±0.59 |
| 120 | 4.44 | 4.24 | 3.31 | 3.38 | 4.10 | 4.79 | 3.64 | 3.56 | 3.93±0.54 |
| 240 | 1.32 | 1.64 | 0.72 | 0.90 | 1.51 | 1.90 | 0.87 | 1.18 | 1.26±0.42 |
| 360 | ND | 1.12 | ND | ND | ND | 0.84 | ND | 0.70 | 0.89±0.21 |
| 480 | ND | 0.43 | ND | ND | ND | ND | ND | ND | 0.43 |
| 600 | ND | ND | ND | ND | ND | ND | ND | ND | / |
| 720 | ND | ND | ND | ND | ND | ND | ND | ND | / |

ND：Not determined
