# Supplementary material for: Establishment of an in vivo analytical method for detecting total anti-UFH activity and pharmacokinetic study in PS and R15 in rats
Source: PLoS One. 2025 Oct 7;20(10):e0333619. doi: 10.1371/journal.pone.0333619 (PMC12503259; doi:10.1371/journal.pone.0333619)
Supplement: S1 File — S1 Table. Standard curve of PS in blank plasma. S2 Table. Standard curve of R15 in blank plasma. S3 Table. The stability of PS plasma sample placed in room temperature (25°C) for 30 min (n = 6). S4 Table. The stability of PS plasma sample freeze-thaw three cycles in −20°C (n = 6). S5 Table. The stability of stock solution of PS for 1 week (n = 6). S6 Table. The stability of R15 plasma sample placed in room temperature (25°C) for 30 min (n = 6). S7 Table. The stability of R15 plasma sample freeze-thaw three cycles in −20°C (n = 6). S8 Table. The stability of stock solution of R15 for 1 week (n = 6). S9 Table. Dilution effects of varying concentrations of plasma samples of PS diluted 2-fold, 5-fold, 10-fold, 20-fold (n = 5). S10 Table. Dilution effects of varying concentrations of plasma samples of R15 diluted 2-fold or 100-fold (n = 5). S11 Table. Pharmacokinetic parameters of intravenous infusion administration with PS (300 U/kg) to individual Wistar rats (n = 6). S11 Table. Pharmacokinetic parameters of intravenous infusion administration with PS (300 U/kg) to individual Wistar rats (n = 6). S12 Table. The plasma concentration of PS after intravenous infusion administration with PS (300 U/kg) to individual Wistar rats. ND: Not determined. S13 Table. Pharmacokinetic parameters of intravenous infusion administration with R15 (2700 U/kg) to individual Wistar rats (n = 8). S14 Table. Pharmacokinetic parameters of intravenous infusion administration with R15 (900 U/kg) to individual Wistar rats (n = 8). S15 Table. Pharmacokinetic parameters of intravenous infusion administration with R15 (300 U/kg) to individual Wistar rats (n = 8). S16 Table. The plasma concentration of R15 after intravenous infusion administration with R15 (300 U/kg) to individual Wistar rats. ND: Not determined. S17 Table. The plasma concentration of R15 after intravenous infusion administration with R15 (900 U/kg) to individual Wistar rats. ND: Not determined. S18 Table. The plasma concentration of [file pone.0333619.s001.zip › S File/S18_File.docx]

**S18 Table. The plasma concentration of R15 after intravenous infusion administration with R15（2700 U/kg）to individual Wistar rats**

| **Time (min)** | **Concentration (μg/mL)** | | | | | | | | **Mean±SD** |
| --- | --- | --- | --- | --- | --- | --- | --- | --- | --- |
|  | **1#** | **5#** | **8#** | **13#** | **21#** | **22#** | **27#** | **28#** |  |
| 0 | 0.00 | 0.00 | 0.00 | 0.00 | 0.00 | 0.00 | 0.00 | 0.00 | 0.00 |
| 1 | 14.61 | 10.94 | 12.70 | 13.51 | 15.63 | 8.27 | 7.46 | 10.11 | 11.65±2.95 |
| 5 | 8.51 | 5.18 | 4.00 | 5.00 | 6.19 | 2.98 | 4.49 | 5.50 | 5.23±1.64 |
| 15 | 4.80 | 2.94 | 4.71 | 3.27 | 6.92 | 3.38 | 6.05 | 5.53 | 4.70±1.43 |
| 30 | 4.14 | 2.69 | 3.27 | 2.35 | 5.02 | 3.23 | 4.48 | 4.14 | 3.67±0.92 |
| 60 | 4.12 | 3.49 | 3.65 | 3.10 | 4.74 | 3.08 | 5.78 | 3.77 | 3.97±0.91 |
| 120 | 4.56 | 2.72 | 3.79 | 2.96 | 3.51 | 3.05 | 3.64 | 3.57 | 3.48±0.58 |
| 240 | 3.67 | 1.52 | 2.82 | 1.86 | 2.40 | 2.27 | 1.41 | 2.81 | 2.34±0.76 |
| 360 | 2.30 | ND | 1.54 | 1.37 | 1.93 | 1.75 | 1.12 | 2.24 | 1.75±0.44 |
| 480 | 1.92 | ND | 0.51 | 1.25 | 1.66 | 1.07 | 0.66 | 1.50 | 1.22±0.52 |
| 600 | 0.83 | ND | ND | 0.62 | 0.97 | 0.74 | ND | 0.68 | 0.77±0.14 |
| 720 | 0.46 | ND | ND | ND | 0.45 | ND | ND | ND | 0.46±0.01 |
| 840 | ND | ND | ND | ND | ND | ND | ND | ND | / |

ND：Not determined
