# Supplementary material for: Establishment of an in vivo analytical method for detecting total anti-UFH activity and pharmacokinetic study in PS and R15 in rats
Source: PLoS One. 2025 Oct 7;20(10):e0333619. doi: 10.1371/journal.pone.0333619 (PMC12503259; doi:10.1371/journal.pone.0333619)
Supplement: S1 File — S1 Table. Standard curve of PS in blank plasma. S2 Table. Standard curve of R15 in blank plasma. S3 Table. The stability of PS plasma sample placed in room temperature (25°C) for 30 min (n = 6). S4 Table. The stability of PS plasma sample freeze-thaw three cycles in −20°C (n = 6). S5 Table. The stability of stock solution of PS for 1 week (n = 6). S6 Table. The stability of R15 plasma sample placed in room temperature (25°C) for 30 min (n = 6). S7 Table. The stability of R15 plasma sample freeze-thaw three cycles in −20°C (n = 6). S8 Table. The stability of stock solution of R15 for 1 week (n = 6). S9 Table. Dilution effects of varying concentrations of plasma samples of PS diluted 2-fold, 5-fold, 10-fold, 20-fold (n = 5). S10 Table. Dilution effects of varying concentrations of plasma samples of R15 diluted 2-fold or 100-fold (n = 5). S11 Table. Pharmacokinetic parameters of intravenous infusion administration with PS (300 U/kg) to individual Wistar rats (n = 6). S11 Table. Pharmacokinetic parameters of intravenous infusion administration with PS (300 U/kg) to individual Wistar rats (n = 6). S12 Table. The plasma concentration of PS after intravenous infusion administration with PS (300 U/kg) to individual Wistar rats. ND: Not determined. S13 Table. Pharmacokinetic parameters of intravenous infusion administration with R15 (2700 U/kg) to individual Wistar rats (n = 8). S14 Table. Pharmacokinetic parameters of intravenous infusion administration with R15 (900 U/kg) to individual Wistar rats (n = 8). S15 Table. Pharmacokinetic parameters of intravenous infusion administration with R15 (300 U/kg) to individual Wistar rats (n = 8). S16 Table. The plasma concentration of R15 after intravenous infusion administration with R15 (300 U/kg) to individual Wistar rats. ND: Not determined. S17 Table. The plasma concentration of R15 after intravenous infusion administration with R15 (900 U/kg) to individual Wistar rats. ND: Not determined. S18 Table. The plasma concentration of [file pone.0333619.s001.zip › S File/S6_File.docx]

**S6 Table. The stability of R15 plasma sample placed in room temperature (25℃) for 30 min (n=6)**

|  | **Concentration (μg/mL)** | |
| --- | --- | --- |
|  | **0.70** | **1.50** |
| Determined value | 0.69 | 1.61 |
|  | 0.68 | 1.62 |
|  | 0.72 | 1.60 |
|  | 0.73 | 1.63 |
|  | 0.70 | 1.79 |
|  | 0.70 | 1.52 |
| n | 6 | 6 |
| Mean | 0.70 | 1.63 |
| SD | 0.02 | 0.09 |
| RSD% | 2.62 | 5.44 |
| RE% | 0.24 | 8.57 |
