# Supplementary material for: Establishment of an in vivo analytical method for detecting total anti-UFH activity and pharmacokinetic study in PS and R15 in rats
Source: PLoS One. 2025 Oct 7;20(10):e0333619. doi: 10.1371/journal.pone.0333619 (PMC12503259; doi:10.1371/journal.pone.0333619)
Supplement: S1 File — S1 Table. Standard curve of PS in blank plasma. S2 Table. Standard curve of R15 in blank plasma. S3 Table. The stability of PS plasma sample placed in room temperature (25°C) for 30 min (n = 6). S4 Table. The stability of PS plasma sample freeze-thaw three cycles in −20°C (n = 6). S5 Table. The stability of stock solution of PS for 1 week (n = 6). S6 Table. The stability of R15 plasma sample placed in room temperature (25°C) for 30 min (n = 6). S7 Table. The stability of R15 plasma sample freeze-thaw three cycles in −20°C (n = 6). S8 Table. The stability of stock solution of R15 for 1 week (n = 6). S9 Table. Dilution effects of varying concentrations of plasma samples of PS diluted 2-fold, 5-fold, 10-fold, 20-fold (n = 5). S10 Table. Dilution effects of varying concentrations of plasma samples of R15 diluted 2-fold or 100-fold (n = 5). S11 Table. Pharmacokinetic parameters of intravenous infusion administration with PS (300 U/kg) to individual Wistar rats (n = 6). S11 Table. Pharmacokinetic parameters of intravenous infusion administration with PS (300 U/kg) to individual Wistar rats (n = 6). S12 Table. The plasma concentration of PS after intravenous infusion administration with PS (300 U/kg) to individual Wistar rats. ND: Not determined. S13 Table. Pharmacokinetic parameters of intravenous infusion administration with R15 (2700 U/kg) to individual Wistar rats (n = 8). S14 Table. Pharmacokinetic parameters of intravenous infusion administration with R15 (900 U/kg) to individual Wistar rats (n = 8). S15 Table. Pharmacokinetic parameters of intravenous infusion administration with R15 (300 U/kg) to individual Wistar rats (n = 8). S16 Table. The plasma concentration of R15 after intravenous infusion administration with R15 (300 U/kg) to individual Wistar rats. ND: Not determined. S17 Table. The plasma concentration of R15 after intravenous infusion administration with R15 (900 U/kg) to individual Wistar rats. ND: Not determined. S18 Table. The plasma concentration of [file pone.0333619.s001.zip › S File/S9_File.docx]

**S9 Table. Dilution effects of varying concentrations of plasma samples of PS diluted 2-fold, 5-fold, 10-fold, 20-fold (n=5)**

|  | **Concentration (μg/mL)** | | | | | | | |
| --- | --- | --- | --- | --- | --- | --- | --- | --- |
|  | **1** | **2-fold** | **1** | **5-fold** | **1** | **10-fold** | **1** | **20-fold** |
| Determined value | 1.00 | 1.99 | 0.83 | 4.17 | 0.96 | 9.6 | 0.98 | 19.5 |
|  | 1.00 | 2.00 | 1.00 | 4.98 | 1.01 | 10.1 | 1.06 | 21.2 |
|  | 1.07 | 2.15 | 1.01 | 5.04 | 1.01 | 10.1 | 1.02 | 20.4 |
|  | 1.01 | 2.03 | 1.03 | 5.16 | 1.04 | 10.4 | 1.03 | 20.7 |
|  | 1.00 | 2.01 | 1.01 | 5.07 | 1.04 | 10.4 | 1.01 | 20.2 |
| n | 5 | 5 | 5 | 5 | 5 | 5 | 5 | 5 |
| Mean | 1.02 | 2.03 | 0.98 | 4.88 | 1.01 | 10.1 | 1.02 | 20.4 |
| SD | 0.03 | 0.07 | 0.08 | 0.41 | 0.03 | 0.3 | 0.03 | 0.6 |
| RSD% | 3.22 | 3.22 | 8.30 | 8.30 | 3.37 | 3.37 | 3.07 | 3.07 |
| RE% | 1.70 | 1.70 | -2.35 | -2.35 | 1.01 | 1.01 | 1.99 | 1.99 |
